# Tandem polymer solar cells: simulation and optimization through a multiscale scheme

**DOI:** 10.3762/bjnano.8.13

**Published:** 2017-01-12

**Authors:** Fanan Wei, Ligang Yao, Fei Lan, Guangyong Li, Lianqing Liu

**Affiliations:** 1 School of Mechanical Engineering and Automation, Fuzhou University, Fuzhou, 350116, China; 2 Department of Electrical and Computer Engineering, University of Pittsburgh, Pittsburgh, PA, USA; 3 State Key Laboratory of Robotics, Shenyang Institute of Automation, CAS, Shenyang, China,; 4 University of Chinese Academy of Sciences, Beijing, China

**Keywords:** genetic algorithm, Monte Carlo simulation, simplex searching, tandem polymer solar cells

## Abstract

In this paper, polymer solar cells with a tandem structure were investigated and optimized using a multiscale simulation scheme. In the proposed multiscale simulation, multiple aspects – optical calculation, mesoscale simulation, device scale simulation and optimal power conversion efficiency searching modules – were studied together to give an optimal result. Through the simulation work, dependencies of device performance on the tandem structures were clarified by tuning the thickness, donor/acceptor weight ratio as well as the donor–acceptor distribution in both active layers of the two sub-cells. Finally, employing searching algorithms, we optimized the power conversion efficiency of the tandem polymer solar cells and located the optimal device structure parameters. With the proposed multiscale simulation strategy, poly(3-hexylthiophene)/phenyl-C61-butyric acid methyl ester and (poly[2,6-(4,4-bis-(2-ethylhexyl)-4*H*-cyclopenta[2,1-*b*;3,4-*b*]dithiophene)-alt-4,7-(2,1,3-benzothiadiazole)])/phenyl-C61-butyric acid methyl ester based tandem solar cells were simulated and optimized as an example. Two configurations with different sub-cell sequences in the tandem photovoltaic device were tested and compared. The comparison of the simulation results between the two configurations demonstrated that the balance between the two sub-cells is of critical importance for tandem organic photovoltaics to achieve high performance. Consistency between the optimization results and the reported experimental results proved the effectiveness of the proposed simulation scheme.

## Introduction

Polymer solar cells, also known as organic solar cells, have been attracting a wealth of attention due to their great potential as an alternative to the presently extensively used inorganic solar cells. However, the wide application of polymer solar cells is still highly restricted by their poor device performance (especially, the low power conversion efficiency (PCE)) when compared with their inorganic counterparts. Therefore, great efforts have been devoted to improving the performance of polymer photovoltaics. To fulfil this goal, various methods, including annealing [1], active materials modification [2] and device structures tuning [3–4], were employed and explored. According to the reports by different research groups, exciting achievements have been witnessed in the past decade [5], among which tandem structures [6–8], created by stacking two single organic solar cells together, have been demonstrated to be one of the most effective solutions.

**Figure 1:**
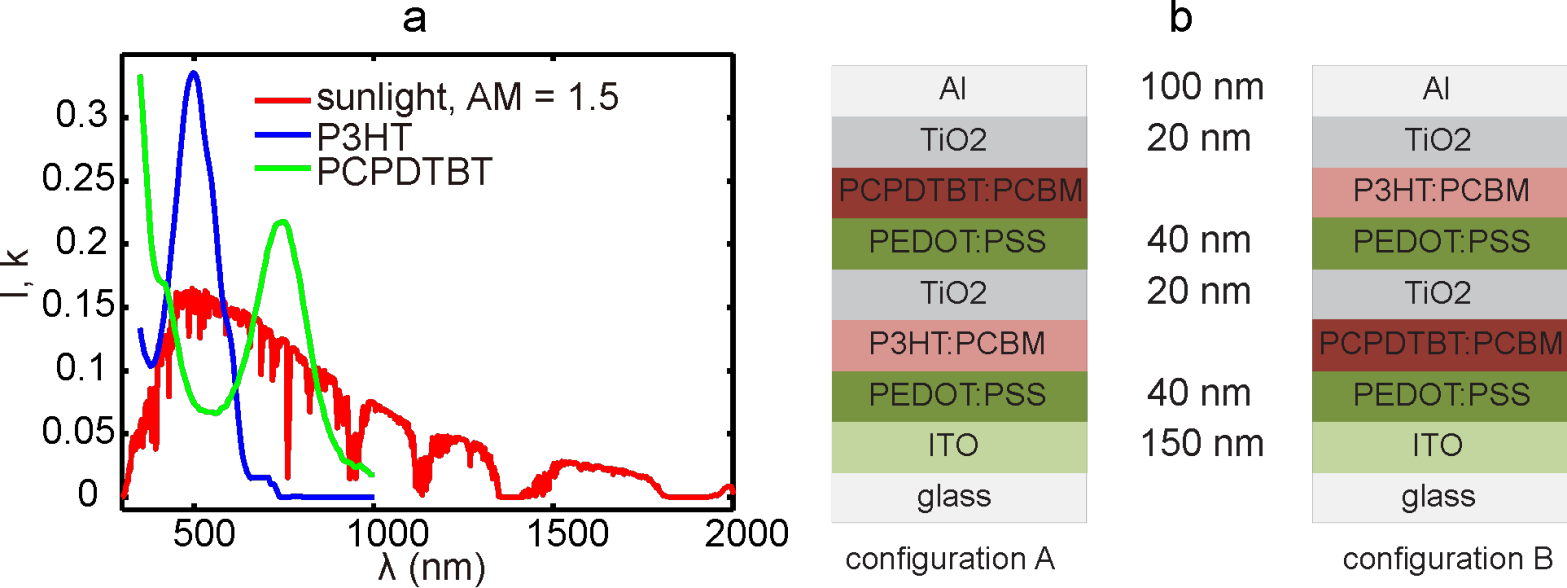
(a) Spectrum of sunlight and different active materials used in tandem organic solar cells. (b) The two configurations (configuration A: left; configuration B: right) of tandem polymer solar cells investigated in this paper.

Due to the unavoidable mismatch between the absorption spectrum of active materials and that of the sunlight, a large portion of the sunlight energy will be lost in organic photovoltaics when a single active material is employed. Thus, as illustrated in Figure 1, the concept of a tandem structure provides a promising solution to this issue by expanding the absorption spectrum using two types of active materials with different band gaps. More and more works [9–11] verify that tandem or triple polymer solar cells can greatly enhance the PCE as compared to single cell devices. However, thus far, structures of tandem polymer solar cells have not been intensively studied given the complexity, and there is still a large margin for the improvement of such devices.

To date, a significant amount of trial-and-error experimental work [7,12] has been conducted to refine tandem device structures, however, experimental work is tedious and far from efficient. Considering the strong optical and electrical coupling between the two sub-cells in tandem photovoltaics, to optimize device performance, it is essential to tune the thickness of both active layers. Furthermore, existing evidence suggests that the distribution of donor/acceptor (D/A) in the active layer is critical in determining the final device performance [1,6]. What also must be considered is that the weight ratio between donor and acceptor is another important factor impacting the PCE of tandem polymer solar cells [12]. Taking all these factors into consideration, the optimization of a tandem structure using trail-and-error experiments can be not only of high cost, but sometimes futile.

Contrary to trail-and-error experiments, simulation is a much more cost-effective tool to tackle this problem. Considering the heavy coupling between the two sub-cells, the simulation and optimization of tandem solar cells are still of great challenge. Even so, some pioneering works on this issue have already been reported [13–19]. Optical coupling between the two sub-cells was investigated and the possible maximum current density through the tandem device was evaluated [16–19]. It was found that the current density cannot be further improved until the photon absorption in the two sub-cells is balanced [16–18]. Some work has been devoted to evaluate the balance of carrier mobility in the two sub-cells [17–18] and even the device performance was estimated according to *J*–*V* curve characteristics constructed through simulations. However, we found that most of the simulation work was focused on tuning the thickness of active layers [20], while efforts were rarely devoted to the optimization of the internal material distribution and D/A weight ratio in the active layers of tandem organic solar cells. The latter, as demonstrated in this work, is critical for optimizing the performance of tandem polymer cells.

In this paper, by taking all the impacting factors into consideration, we mimic the photocurrent generation process and subsequently optimize the device structures in tandem polymer solar cells with poly(3-hexylthiophene)/phenyl-C61-butyric acid methyl ester (P3HT/PCBM) and (poly[2,6-(4,4-bis-(2-ethylhexyl)-4*H*-cyclopenta[2,1-*b*;3,4-*b*]dithiophene)-alt-4,7-(2,1,3-benzothiadiazole)])/PCBM (PCPDTBT/PCBM). The *J*–*V* curves of the device were acquired through a multiscale simulation scheme. Then performance indices were evaluated and related to the thickness, inner morphologies and the weight ratio of the active layers. Finally, using a simplex searching algorithm and genetic algorithm (GA), a global optimal PCE value was found and the corresponding device parameters were obtained. In order to prove the viability of our proposed simulation approach, two different configurations of the tandem devices, as shown in Figure 1b, were both tested and compared with each other.

## Multiscale Simulation of Tandem Polymer Solar Cells

**Figure 2:**
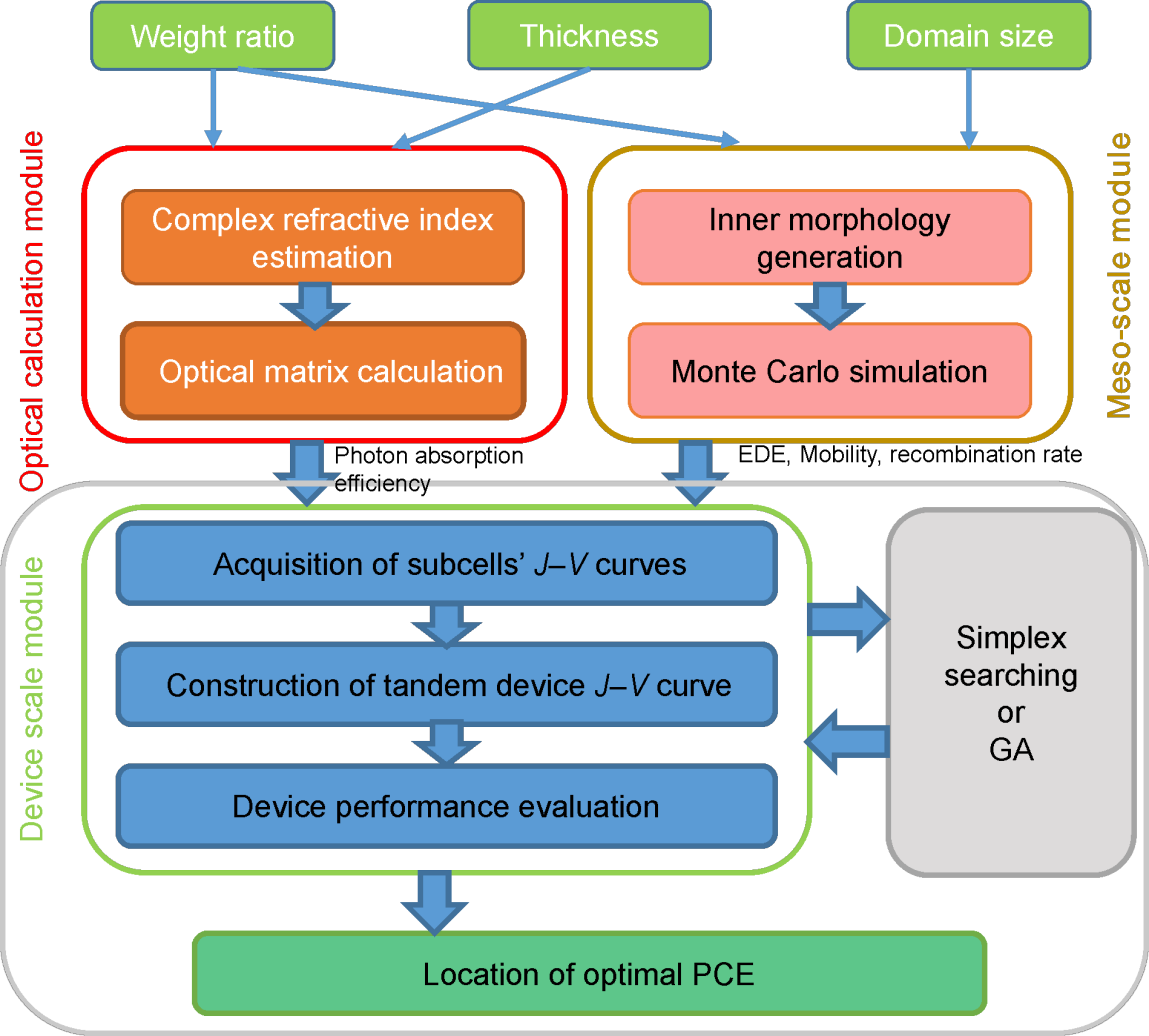
Schematic of the proposed simulation strategy for investigation and optimization of organic solar cells.

To simulate the photocurrent generation process and subsequently evaluate the performance indices for tandem photovoltaics, we designed and realized a simulation scheme and denoted it as multiscale simulation. As illustrated in Figure 2, the schematic of the proposed multiscale simulation was divided into four parts: optical calculation module, mesoscale simulation module, device scale simulation module, and finally, optimal PCE searching module.

### Optical decoupling in optical calculation module

In tandem polymer solar cells, the two stacked sub-cells are optically highly coupled. Thus, an optical decoupling process is required before any evaluation of device performance.

In the optical calculation part developed here, the complex refractive index of the blend with varied D/A weight ratios (2:1, 3:2, 1:1, 2:3 and 1:2) in the P3HT/PCBM layer was calculated using bulk effective medium method [21]. The optical parameters for pure P3HT and PCBM were provided by McGehee et al. [22]. In the reported experiments [8,23–24], the D/A weight ratio of PCPDTBT/PCBM in polymer solar cells has been varied between 2:1 and 1:4. Due to the lack of optical index for pure PCPDTBT, the weight ratio of PCPDTBT/PCBM was fixed to be a reasonable value of 1:2 in the following simulation work. For all other materials besides the active materials, the complex refractive indices were acquired from [25]. Then, with the optical indices of all the materials in the system known, optical transfer matrix theory [26] was adopted to evaluate the photon absorption efficiency in active layers of the two sub-cells. By conducting the calculation under varied active layer thicknesses (from 1 nm to 400 nm with an interval of 1 nm) and weight ratios, the dependencies of the photon absorption efficiency on the device structures were clarified. Meanwhile, the thickness of the other layers in the device was fixed to the values shown in Figure 1b. Herein, the influence of the inner distribution of the blended materials were ignored because, to our best knowledge, there is no strong evidence to support the contribution of internal morphology to the photon absorption in active layers.

### Bridging macroscale and microscale through mesoscale simulation

As discussed above, the distribution of material at the microscale also affects device performance. To quantize this affect, we need to bridge the gap between microscale and macroscale in the simulation system. In our previous work [27], a mesoscale simulation, i.e., a Monte Carlo (MC) simulation, has been developed to meet this requirement. At the mesoscale, with the weight ratio and average domain size as input parameters, MC simulations were carried out to compute the effective charge carrier mobility and recombination rate. Firstly, the internal morphology in active layers was generated through a simulated annealing method [27]. In the simulated annealing method, the Ising model is adopted to generate the morphology with desired donor–acceptor distribution. Details of the morphology generation process is available in Supporting Information File 1. The simulated morphology agrees well with that acquired from actual experiments (Supporting Information File 1, Figure S1). Then, the distribution of donor and acceptor materials in the blend was quantized using the average domain size (*a*) defined in [27]. A series of morphologies with domain size ranging from 6 nm to 20 nm were prepared. The interval of domain size between adjacent morphologies was 2 nm. The lattice size in the generated morphologies was set to be 3 nm; and the size of the generated morphologies was 180 × 180 × 90 nm. The thickness of the active layers was fixed to be 90 nm in this work for simplification. Using the generated morphologies, generation, transport, recombination and extraction of charge carriers were mimicked and recorded in the MC simulation. The MC simulation was realized through the first reaction method (FRM) (the flow chart of FRM is presented in Supporting Information File 1, Figure S2). The details of FRM are illustrated in Supporting Information File 1. Based on the simulation of exciton transportation, dissociation and extinction processes, exciton dissociation efficiency (EDE, defined in Equation 1) was obtained, which is crucial for the calculation of charge carrier generation rate in the active layers. Electron and hole mobility was related to the average domain size and the electric field in the active layers. Since charge carrier recombination is the main cause of energy loss in organic solar cells, both the bimolecular and monomolecular recombination rate were evaluated (defined as in Equation 2 and Equation 3) and related to the domain size and electric field. A monomolecular recombination event was defined as the recombination between one free carrier and one carrier stuck in traps or dead ends in the active layer, while other recombination events were classified as bimolecular. Then, we varied the D/A weight ratios in the P3HT/PCBM active layer and repeated the mesoscale simulations. For more details of the MC simulation adopted here, please refer to [27]. Some of the parameters used in the MC simulation are presented in Table 1 (see below).

[1]
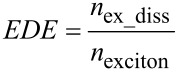


where, *n*_ex_diss_ and *n*_exciton_ are the number of dissociated excitons and the total number of generated excitons in the simulation, respectively. The recombination rates are

[2]
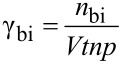


[3]
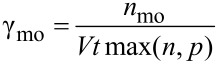


where, *n*_bi_ and *n*_mo_ are the numbers of recombined electron–hole pairs through bimolecular and monomolecular recombination routes, respectively. *V* denotes the total volume of the studied domain and *t* is the elapsed time. *n* and *p* are the concentration of electrons and holes in the system.

### Device performance evaluation through device scale simulation

At the device scale, simulation can give a full picture of the system by considering boundary conditions. Thus, it is reliable to calculate the device performance for tandem solar cells at the device scale.

The photon absorption efficiency, EDE, carrier mobility and recombination rate were inputs into the device scale simulation module [25] and *J*–*V* curves of each single sub-cell were acquired through solving drift–diffusion equations. Device scale simulation was performed for both sub-cells separately. The critical parameters used in the device scale simulation are listed in Table 1. Then, the *J*–*V* curve of the tandem device was constructed by matching the current density of the two sub-cells. Since the two sub-cells are in series, the current density through each of the sub-cells should be the same. The search for the identical current density point was realized by increasing the applied voltage on one sub-cell and decreasing the voltage on the other one by the same amount, while keeping the total applied voltage constant. Applying the same rule, the whole *J*–*V* curve was acquired by going through all the voltage points.

**Table 1:** The parameters used in the multiscale simulation.

Symbol	Value	Unit	Notes

*T*	298	K	temperature
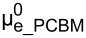	0.02 [28]	cm^2^/(Vs)	local electron mobility in PCBM
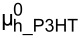	0.003 [29]	cm^2^/(Vs)	local hole mobility in P3HT
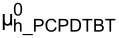	0.1 [24]	cm^2^/(Vs)	local hole mobility in PCPDTBT
*E*_g_P3HT_	1.05 [25]	eV	effective band gap of P3HT/PCBM
*E*_g_PCPDTBT_	1.0 [16]	eV	effective band gap of PCPDTBT/PCBM

### Tandem polymer solar cell optimization through searching algorithms

To the best of our knowledge, the optimization of tandem polymer solar cells were most simply conducted by traversing the whole space in the work reported in [16–20]. Taking the high complexity into account, the computational load would be too high if accurate optimization results are required for tandem polymer solar cells.

For the purpose of relieving computational load and optimizing tandem device structures with all the structure parameters considered, we employed two searching algorithms: simplex searching [30] and GA [31]. In simplex searching, five points were randomly generated in the studied domain, where each point contained all the four investigated variables: thickness (*d*) and domain size (*a*) of the two active layers (*d*_P3HT/PCBM_, *d*_PCPDTBT/PCBM_, *a*_P3HT/PCBM_, *a*_PCPDTBT/PCBM_). Then, during the searching process, the points were updated according to the schematic and flow chart (Figure S3 and S4, in Supporting Information File 1). On the other hand, for GA searching, six samples (six points) with four traits (the four variables) were prepared. During each iteration, the best two samples were selected as the parent ones. The child samples were generated using the two parent samples through inheritance, crossover and mutation operations, as illustrated in Supporting Information File 1, Figure S5. Through both algorithms, the searching process was conducted for different D/A weight ratios. The detailed illustration of the two optimization methods are presented in Supporting Information File 1.

## Results and Discussion

**Figure 3:**
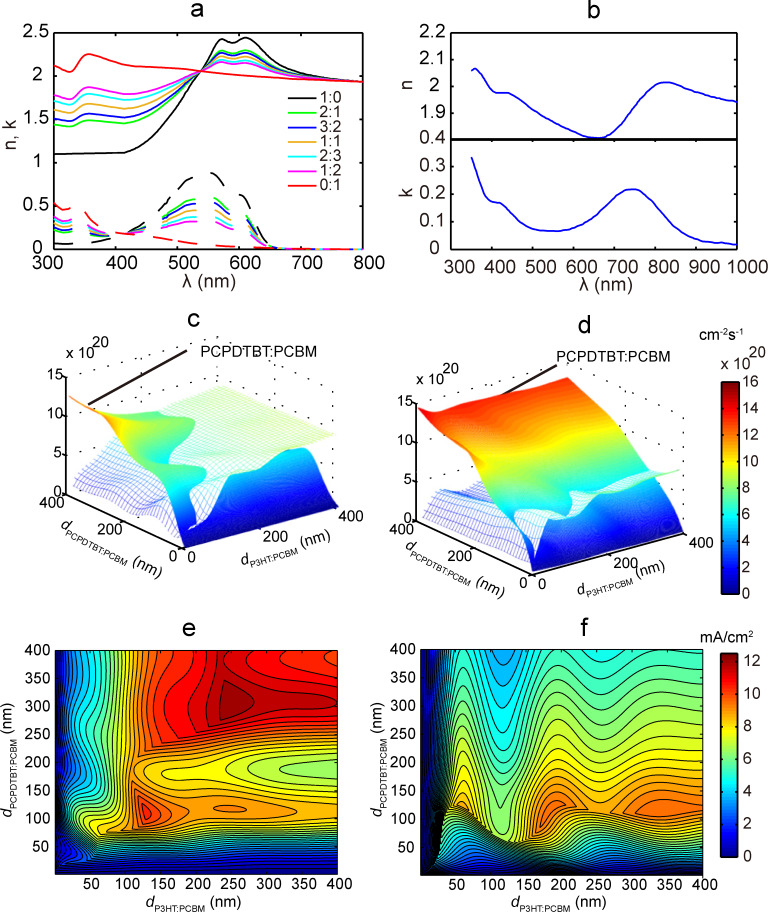
Optical parameters and simulation results from the optical calculation module. The complex refractive index of the P3HT/PCBM blend (a) with different D/A weight ratio (the solid lines are for *n*, and the dashed lines are for *k*) and PCPDTBT/PCBM (b) with weight ratio of 1:2. (c) and (d) (sharing the same color code scale) present the photon absorption efficiency calculated for configuration A and B, respectively. The weight ratios of P3HT/PCBM and PCPDTBT/PCBM active layers are 1:1 and 1:2. The maximum photocurrent estimated according to the photon absorption efficiency for the two device configurations is displayed in (e) and (f) (e and f share the same color code scale).

Through the bulk effective medium approach, the complex refractive index for the P3HT/PCBM blend with different weight ratios was calculated and presented in Figure 3a. As shown in Figure 3b, refractive index for PCPDTBT/PCBM blend was acquired from [25] . From the optical calculation module, the photon absorption efficiency (Figure 3c,d) of the two active layers were computed under varied thickness values for the two layers. Then, as presented in Figure 3e,f, the possible maximum short circuit current density (*J*_sc_) through the device was estimated by assuming that all the absorbed light energy will contribute to the final electric energy with no loss. From Figure 3, it is obvious that configuration A has a much better balance between the two sub-cells than configuration B. Therefore, the possible current density was estimated to be much higher for the configuration with P3HT/PCBM sub-cell at the front of the incident light. For all the estimated maximum current density maps corresponding to the case of different P3HT/PCBM weight ratios under different configurations, please refer to Supporting Information File 1, Figure S6.

**Figure 4:**
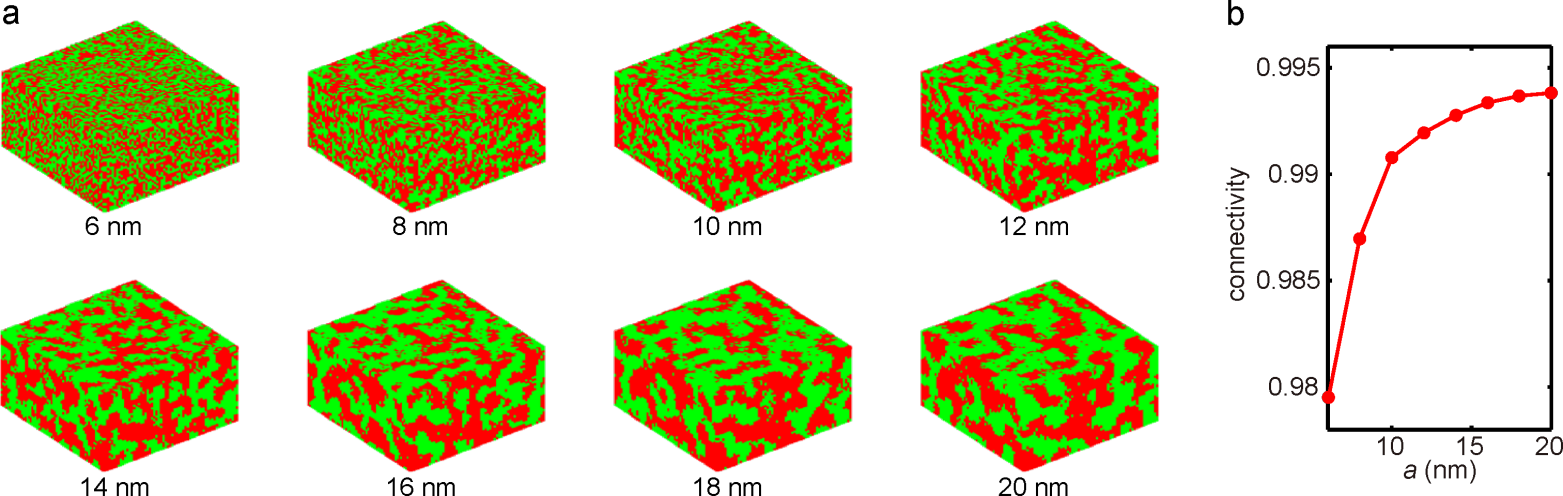
(a) Morphologies generated for 1:1 P3HT (green)/PCBM (red) and (b) the dependence of the connectivity ratio on the domain size for the morphologies in (a).

**Figure 5:**
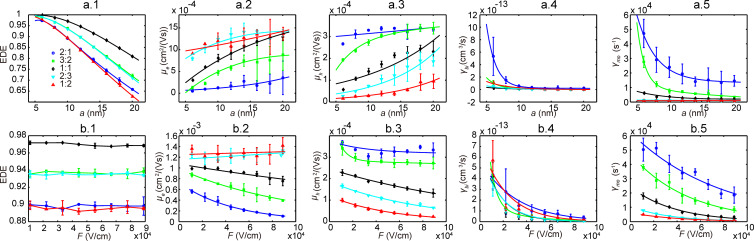
MC simulation results for P3HT/PCBM active layer with different D/A weight ratios. (a.1–5) presents the EDE, electron and hole mobility, bimolecular and monomolecular recombination rate with respect to domain size. (b.1–5) presents the results with respect to electric field.

**Figure 6:**
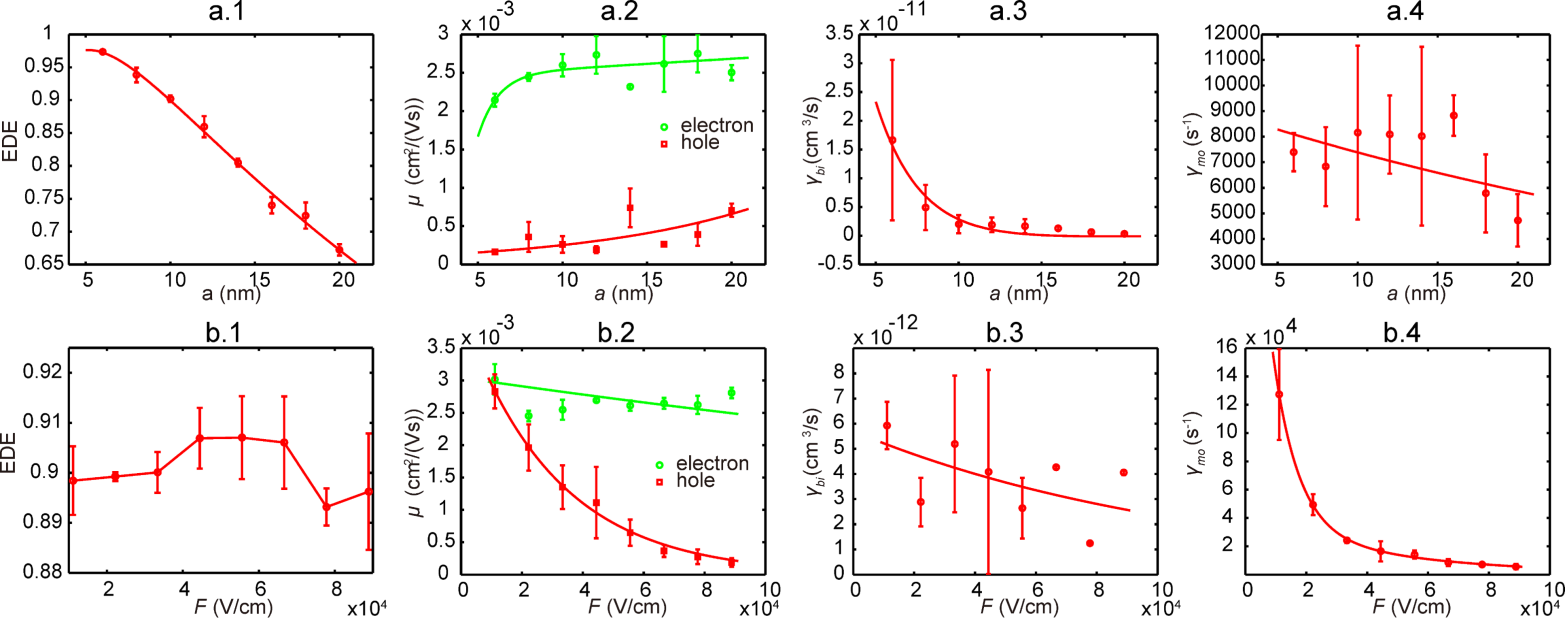
MC simulation results for PCPDTBT/PCBM (1:2) active layer. (a.1–4) presents the EDE, carrier mobility, bimolecular and monomolecular recombination rate with respect to domain size. (b.1–4) presents the results with respect to electric field.

In the mesoscale simulation, knowledge of morphology is required before MC simulations can be performed. For each weight ratio value, three series of morphologies were generated. A series of morphologies for 1:1 P3HT/PCBM are shown in Figure 4a. The connectivity ratio (as defined in [21]) for one series of morphologies of 1:1 P3HT/PCBM is related to the domain size and displayed in Figure 4b. Through the MC simulation, we evaluated EDE, carrier mobility, recombination rate, and also clarified their dependence on domain size and electric field; corresponding plots of these data are shown in Figure 5 and Figure 6. At each data point, the simulations were repeated three times representing the three different morphologies at each domain size and at each weight ratio value. As shown in Figure 5 and Figure 6, EDE decreases with domain size, but keeps almost constant with electric field. Since excitons can only dissociate into electrons and holes at the interface between donor and acceptor, EDE is expected to decrease with domain size because the D/A interface area reduces as domain size increases. On the other hand, excitons are neutral and immune to the external and internal electric field; this explains our observation of independence of EDE on electric field. As indicated from Figure 4b, the connectivity ratio increases with the domain size. Then, as domain size increases, it will be easier for charge carriers to transport through the blend, and more difficult for carriers to recombine with each other. The corresponding results, shown in Figure 5a.2–5 and Figure 6a.2–4, correlate well with the assumptions as discussed above. As observed from Figure 5b.2–5 and Figure 6b.2–4, both the evaluated mobility and recombination rate tend to decrease with the electric field. An appropriate explanation is that many of the carriers can be captured in dead ends or traps because of the complex inner structures in the blend. In such a case, the high electric field will reduce the possibility for the trapped carriers to jump out from the traps, which leads to a lower effective carrier mobility. However, since electrons and holes will be dragged towards the opposite side by the electric field, a higher electric field will guarantee the separation of electrons and holes and subsequently leads to lower recombination rate, both bimolecular and monomolecular.

**Figure 7:**
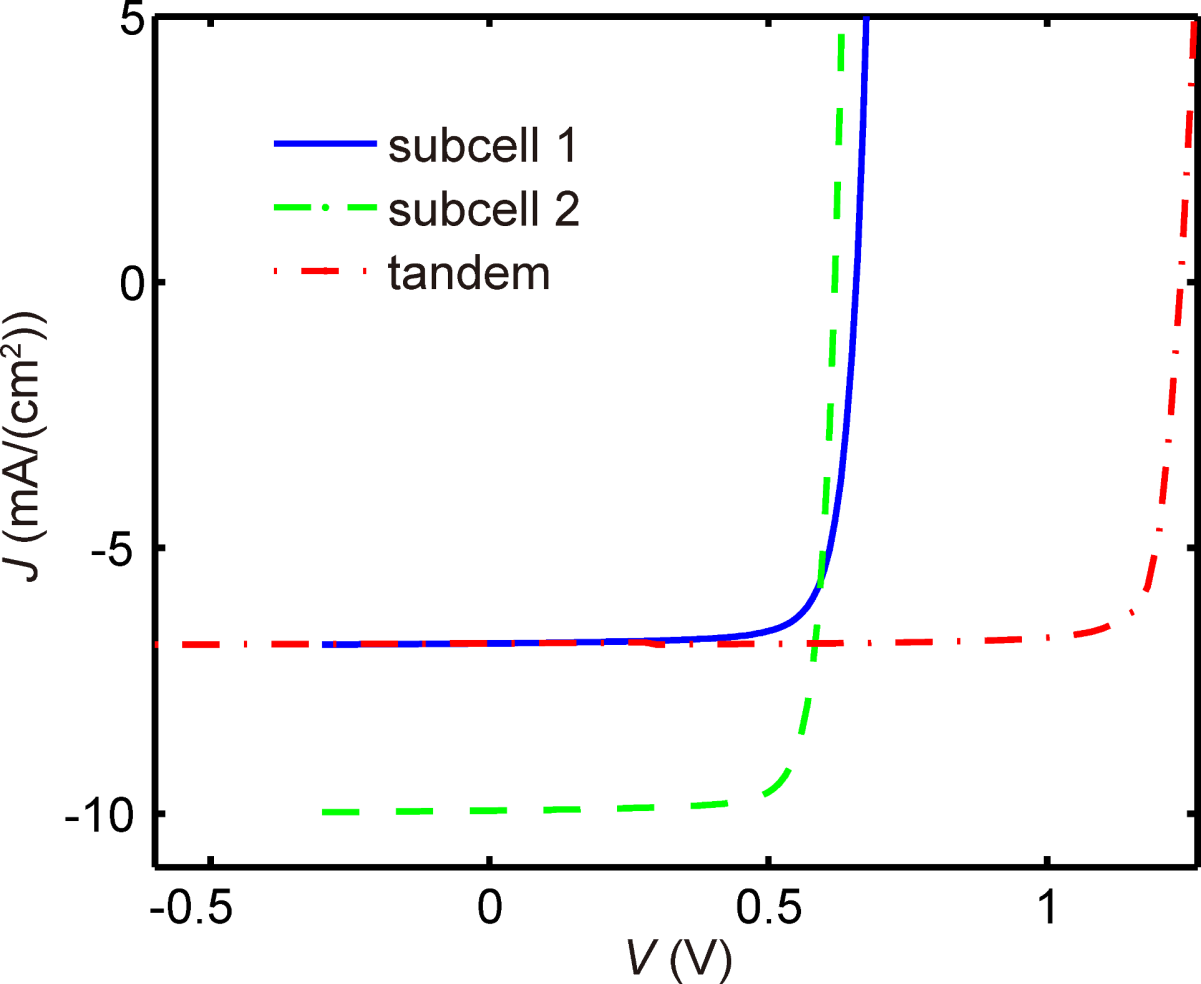
One example of a *J*–*V* curve for a tandem structure constructed from *J*–*V* curves of sub-cells. Sub-cell 1 is based on P3HT/PCBM, while sub-cell 2 is based on PCPDTBT/PCBM.

**Figure 8:**
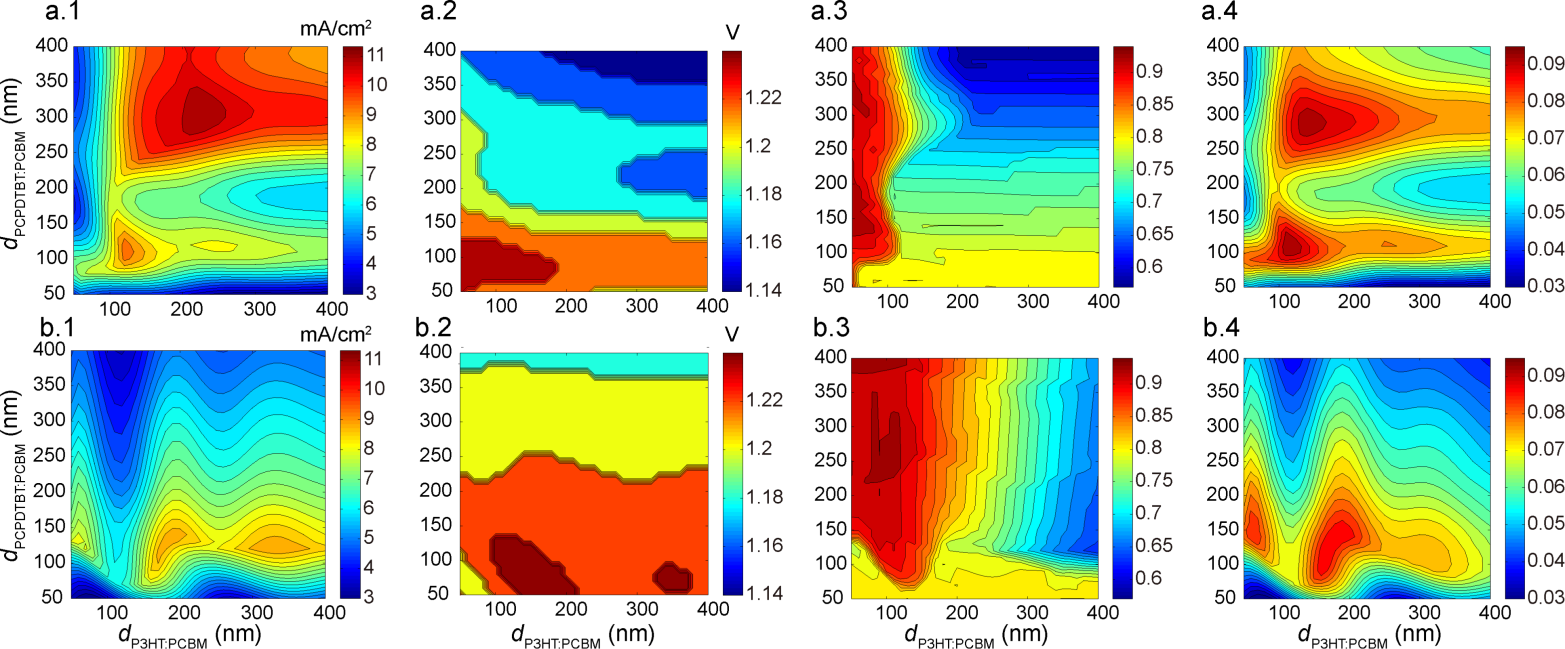
Device performance calculated through the multiscale simulation for configuration A (a) and B (b). a.1 and b.1 show *J*_sc_ with respect to thickness. a.2 and b.2 present *V*_oc_. FF is given in a.3 and b.3. PCE is presented in a.4 and b.4. Here, the weight ratios for P3HT/PCBM and PCPDTBT/PCBM are 1:1 and 1:2, respectively.

The device performance indices were calculated from the tandem device’s *J*–*V* curve, constructed from the *J*–*V* curves of the two sub-cells (illustrated in Figure 7). With the domain size fixed at 10 nm for both active layers, we acquired the device performance indices with respect to active layer thickness changing from 50 nm to 400 nm with an interval of 10 nm. As demonstrated in Figure 8, *J*_sc_, open circuit voltage (*V*_oc_), fill factor (FF) and PCE are obtained for both the two configurations investigated. Considering the independence of photon absorption efficiency on the active layer thickness, the agreement between the *J*_sc_ map (as shown in Figure 8a.1 and 8b.1) and the map of possible maximum current shown in Figure 3e,f, suggests that the *J*_sc_ of tandem polymer solar cells is largely determined by the photon absorption efficiency. Smaller thickness means higher electric field in the device; higher electric field leads to lower recombination rate, which results in higher *V*_oc_ and FF. These discussions are in conjunction with the results presented in Figure 8a.2,3 and 8b.2,3. For different weight ratios, the PCE values are all estimated and their dependence on the active layer thickness and domain size are presented in Supporting Information File 1, Figure S7 and Figure S8, respectively.

**Figure 9:**
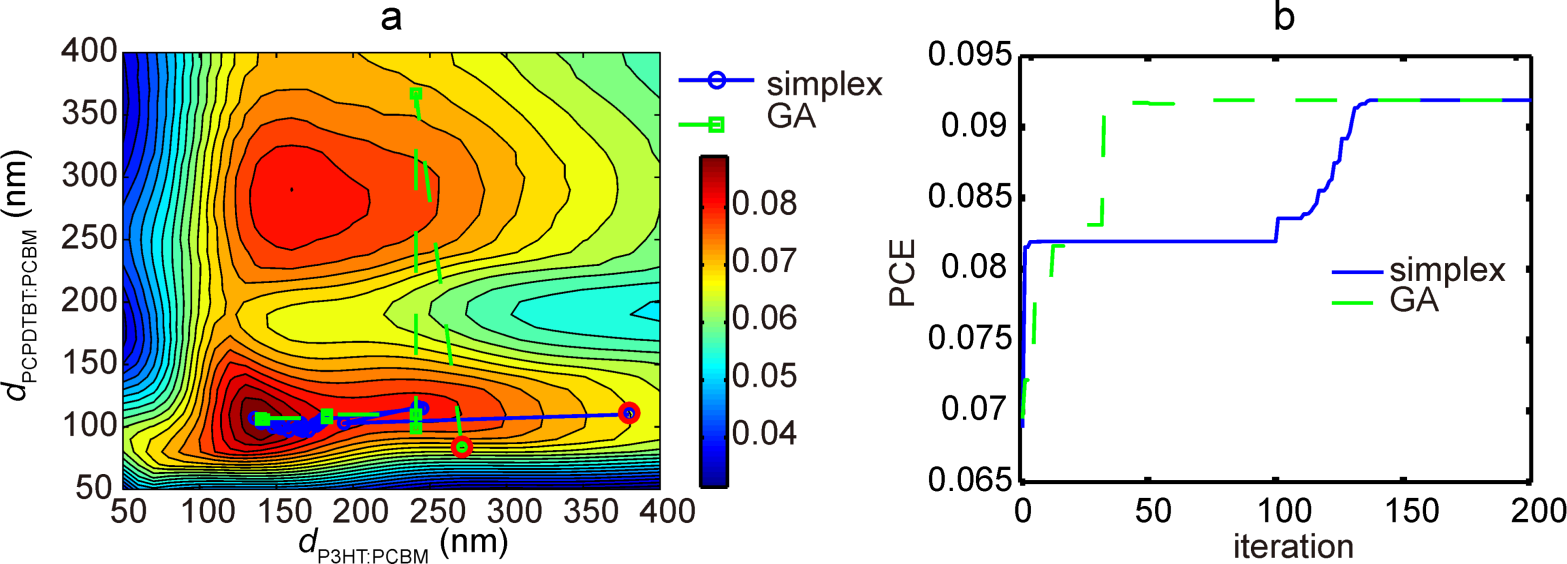
(a) Presents the evolution of optimized active layer thicknesses during the search of the optimal PCE value. Both the simplex searching algorithm and GA are tested. The random start points are indicated by the red circles. The corresponding PCE value evolutions are displayed in (b). Here, the weight ratios are 1:2 for both P3HT/PCBM and PCPDTBT/PCBM.

Next, the searching algorithms were employed to locate the optimal PCE value with respect to thickness, domain size and weight ratio of the two active layers. In order to check the effectiveness of the searching algorithms, tests were performed with domain size and weight ratio values fixed. The starting points, as indicated in Figure 9a using red circles, were generated randomly. As the number of searching iterations increases, both simplex searching method and GA reach the same optimal PCE value with the location (P3HT/PCBM layer thickness of 141 nm, PCPDTBT/PCBM layer thickness of 106 nm). Both algorithms find the PCE peak value of 0.919, which proves the effectiveness of the searching methods in tackling this issue. Additionally, while traversing the whole space to calculate all the PCE values takes over 24 hours, the optimization algorithms acquired the optimal PCE value within 10 minutes. On the other hand, GA is expected to jump out of local optimal PCE because of the mutation operation in the child sample generation process, while the simplex method tends to be easily trapped at the local peak.

**Figure 10:**
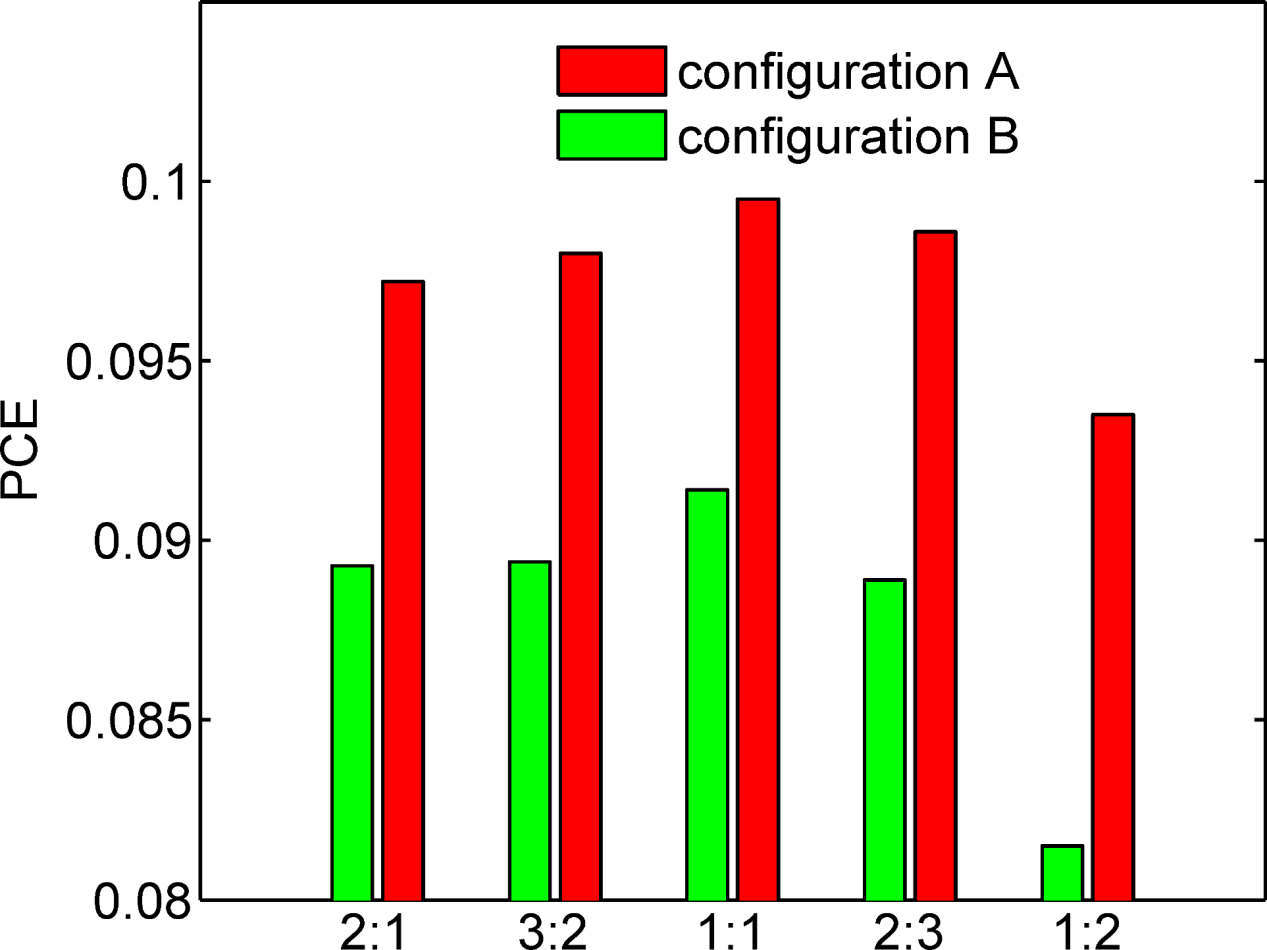
Optimal PCE values for different configurations and different D/A weight ratios in the P3HT/PCBM blend.

Finally, the optimization of a tandem structure with all active layer parameters (*d*_P3HT/PCBM_, *d*_PCPDTBT/PCBM_, *a*_P3HT/PCBM_, *a*_PCPDTBT/PCBM_, and η_P3HT/PCBM_ (D/A ratio in P3HT/PCBM active layer)) taken into consideration was conducted using the two searching algorithms as discussed above. By repeating the searching behaviour at least ten times for each P3HT/PCBM weight ratio value using both of the algorithms, we get the initially optimized PCE values. Then, to check whether the PCE values were globally optimized, the corresponding locations for the initial optimal PCE values were delivered into both algorithms as one of the starting points. The acquired PCE values were recognized as the global peak until no further increase was observed when both of the algorithms were performed. The final optimization results are listed in Table 2. As indicated by Table 2 and Figure 10, the configuration with P3HT/PCBM as the front sub-cell facing the incident light achieves relatively better performance than the configuration with PCPDTBT/PCBM at the front. As a lower band gap, active material, PCPDTBT has considerably stronger optical absorption than P3HT. Thus, if the sub-cell with PCPDTBT is put first, a large portion of light energy will be harvested by the PCPDTBT based sub-cell. Therefore, a significant unbalance, as indicated from Figure 3d,f, between the two sub-cells will be evidenced, which ruins the device performance in configuration B. Then, the tandem structure of configuration B will attempt to counteract the unbalance by increasing the thickness of the P3HT/PCBM layer and simultaneously reducing the PCPDTBT/PCBM layer thickness. Therefore, in configuration B, *d*_P3HT/PCBM_ is much higher than *d*_PCPDTBT/PCBM_ in the optimized structure. As shown in Table 2, the PCE champion is configuration A with 1:1 P3HT/PCBM; and the optimal PCE is almost 10%. For almost all configurations and weight ratios, the optimized device is located with active layer thickness between 100 nm and 200 nm and an average domain size between 6 nm and 11 nm. A thick active layer can ensure high current density, but meanwhile leads to serious recombination and low FF. On the other hand, lower domain size will lead to higher EDE, but also lower carrier mobility and higher recombination rate. Therefore, thickness and domain size should be tuned to proper values in optimized device. As revealed from the results, the tandem device benefits from much more than it suffers from low domain size and the optimal active layer thickness values are in line with those of most reported PCE works [7–8,13,15].

**Table 2:** Optimized device parameters of both the two configurations in a tandem structure studied herein.

Devices		*d*_P3HT/PCBM_ (nm)	*d*_PCPDTBT/PCBM_ (nm)	*a*_P3HT/PCBM_ (nm)	*a*_PCPDTBT/PCBM_ (nm)	PCE

A	2:1	120	110	7	7	0.0972
	3:2	120	110	7	6	0.0980
	**1:1**	**120**	**108**	**8**	**7**	**0.0995**
	2:3	130	108	9	6	0.0986
	1:2	150	106	10	7	0.0935

B	2:1	64	144	6	8	0.0893
	3:2	171	94	10	7	0.0894
	**1:1**	**170**	**107**	**10**	**7**	**0.0914**
	2:3	172	91	10	6	0.0889
	1:2	175	78	11	7	0.0815

Due to the lack of data for the refractive index of pure PCPDTBT polymer, we have fixed the D/A weight ratio in the PCPDTBT/PCBM layer to be 1:2. However, as demonstrated in Supporting Information File 1, Figure S7 and S8, the D/A weight ratio of the active layer impacts the PCE value significantly. Therefore, the performance of the tandem device investigated is to be further optimized with the D/A weight ratio of PCPDTBT/PCBM layer varied.

## Conclusion

In summary, we have proposed a multiscale simulation and have demonstrated its capability in the simulation and optimization of tandem organic solar cells. Both optical and electric coupling between the two stacked sub-cells were overcome through our optical calculation module and the device scale simulation module, respectively. The performance criteria of the tandem solar cells based on P3HT, PCPDTBT and PCBM were evaluated through the simulation work and related to the thickness, domain size and D/A weight ratios in the active layers. Finally, we found the optimal device structure parameters through both simplex algorithm and GA. The optimized tandem device parameters were in agreement with those reported in fabricated devices. This work not only provides promising guidance for tandem polymer solar cell fabrication, but also sheds light on the fundamental principles of tandem photovoltaic devices. Additionally, this approach can be easily applied to optimizing tandem solar cells based on other materials as long as the refractive index and local carrier mobility of the new materials are available.

## Supporting Information

Comparison between experimental morphology and simulated morphology, the flow chart of FRM, the illustration of simplex searching algorithm and GA. Additional simulation results, including the projected current density and PCE with respect to active layer thickness and active layer domain size.

File 1 Additional simulation results and information used for calculations.

File 2 MATLAB code for the morphology generation process.
