# Supplementary material for: Tandem polymer solar cells: simulation and optimization through a multiscale scheme
Source: Beilstein J Nanotechnol. 2017 Jan 12;8:123–33. doi: 10.3762/bjnano.8.13 (PMC5238685; doi:10.3762/bjnano.8.13)
Supplement: File 1 — Additional simulation results and information used for calculations. [file Beilstein_J_Nanotechnol-08-123-s001.pdf]

Supporting Information
for
Tandem polymer solar cells: simulation and optimization through a multiscale scheme

Fanan Wei[1], Ligang Yao[1], Fei Lan[2], Guangyong Li*[2] and Lianqing Liu*[3,4]

Address: [1]School of Mechanical Engineering and Automation, Fuzhou University, Fuzhou, China, 350116, [2]Department of Electrical and Computer Engineering, University of Pittsburgh, Pittsburgh, PA, USA, [3]State Key Laboratory of Robotics, Shenyang Institute of Automation, CAS, Shenyang, China, and [4]University of Chinese Academy of Sciences, Beijing, China

Email: Guangyong Li - gul6@pitt.edu; Lianqing Liu - lqliu@sia.cn

* Corresponding author

Additional simulation results and information used for calculations

1. Donor/acceptor blend morphology generation through simulated annealing approach

For the structure model generation part, we employed Ising model [1] with simulated annealing method [2]. The detailed generation process for the active (heterojunction) layer is discussed as follows.

First, the active layer is discretized into small cubes with a lattice constant of 3 nm. Every cubes, also called site, is assigned a value 0 or 1 with probability determined by the weight ratio between donor and acceptor.

Second, choose two neighboring sites randomly from all the lattices and calculate the Ising Hamiltonian value of the system comprised of the chosen two sites, their neighbors, and second nearest neighbors according to the following equation (Eq. S1).

$$\varepsilon_i = -\frac{J}{2}\sum_j(\delta_{si,sj} - 1) \qquad \text{(S1)}$$

where, $\delta_{si,sj}$ is the delta function, and $J - k_B T$; the contribution of the second-nearest neighbors is scaled by a factor of $1/\sqrt{2}$.

Third, exchange the spin value (0 or 1) of the chosen two sites with the probability:

$$P(\Delta\varepsilon) = \frac{\exp(-\Delta\varepsilon/k_B T)}{1+\exp(-\Delta\varepsilon/k_B T)} \qquad \text{(S2)}$$

where $\Delta\varepsilon$ is the difference between Hamiltonian values before and after the exchange.

Finally, after a great number of attempted spins exchanges, a morphology series with the required domain sizes is generated. Domain size value is estimated through Eq. S3.

$$\mathrm{d} = 6 * \min(\alpha, 1 \quad \alpha) * \mathrm{V}/\mathrm{A} \qquad \text{(S3)}$$

where α is the ratio of one component (either donor or acceptor), A is the donor/acceptor interfacial area, and V represents the volume of the bulk.

With the purpose of clarification, MATLAB code for the morphology generation process is presented in Supporting Information File 2.

In order to compare the simulated and actual active layer morphology, we prepared P3HT:PCBM blend with 1:1 weight ratio and characterized the surface of the blend using AFM. The AFM phase image is separated according to the threshold determined by the

weight ratio between donor and acceptor. The separated experimental morphology is presented at the left of Fig.S1. Cross section image of the simulated active layer is displayed in the right sub-figure of Fig. S1. As indicated from the comparison shown in Fig. S1, the experimental result agrees relatively well with the blended morphology generated from Ising model.

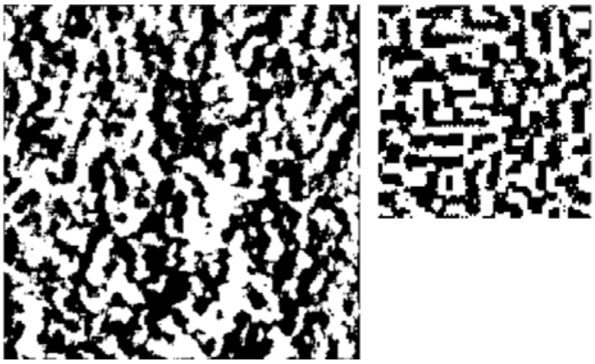

Figure S1: Comparison of the surface image (500 nm*500 nm) obtained from experimental data (left) and the cross section morphology image (300 nm*300 nm) generated from simulation (right) for P3HT:PCBM with weight ratio of 1:1.

2. Monte Carlos simulation module

Monte Carlo (MC) simulation is performed to mimic the photocurrent generation process in the organic solar cells. Three types of carriers, excitons, electrons and holes, are taken into consideration in the MC simulation. First, an exciton is generated at a random site in the donor-acceptor (P3HT:PCBM or PCPDTBT:PCBM) heterojunction layer. The generated exciton will dissociate into separated into electron and hole when it transport to the donor/acceptor interface. The generated free electron (hole) travels through the heterojunction layer in donor(acceptor) under the electric field originated from a combination of external voltage, electrostatic interactions among carriers and built-in potential. Charge carriers are extracted and contribute to the photocurrent when they are neighboring to their corresponding electrode. At the same time, electron and hole could also decay through recombining with each other at the D/A interface. In order to implement the MC simulation, the so-called First Reaction Method (FRM) (Flow chart of FRM is illustrated in Fig. S2) is adopted. For more information, please refer to Ref. [3].

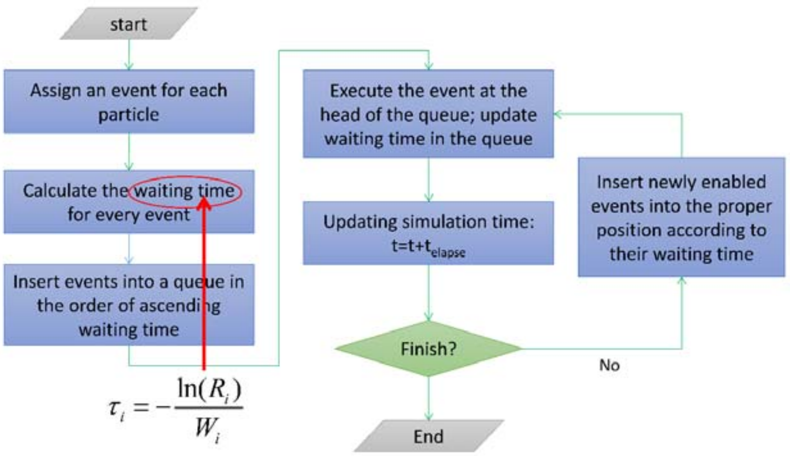

Figure S2: Flow chart of the FRM strategy adopted in this paper.

3. Simplex searching algorithm for tandem polymer solar cells optimization

For tandem polymer solar cells, the thickness, domain size and D/A weight ratio of both the two active layers affect the final performance of the device. Therefore, there are five variables ($d_{P3HT:PCBM}$, $d_{PCPDTBT:PCBM}$, $a_{P3HT:PCBM}$, $a_{PCPDTBT:PCBM}$, $\eta_{P3HT:PCBM}$ (D/A weight ratio of P3HT:PCBM)) to be optimized in this work. Since $\eta_{P3HT:PCBM}$ is limited to be several discretized values (2:1, 3:2, 1:1, 2:3, 1:2), the variable number is decreased into four. Considering the high complexity of the problem, we employed optimization algorithms (simplex searching and GA) to conduct the optimization process.

In the case of simplex searching algorithm, five points (X_i, i = 1~5) are generated in the studied space. Each point is composed of all the four variables ($d_{P3HT:PCBM}$, $d_{PCPDTBT:PCBM}$, $a_{P3HT:PCBM}$, $a_{PCPDTBT:PCBM}$, $\eta_{P3HT:PCBM}$). For example, one point (100, 120, 12, 16) represents: thickness of P3HT:PCBM active layer $d_{P3HT:PCBM}$ = 100 nm, thickness of PCPDTBT:PCBM active layer $d_{PCPDTBT:PCBM}$ = 120 nm, domain size of P3HT:PCBM layer $a_{PCPDTBT:PCBM}$ = 12 nm, domain size of PCPDTBT:PCBM layer $a_{PCPDTBT:PCBM}$ = 16 nm. The PCE values ($f(X_i)$, i = 1~5) corresponding to the five initial points are calculated through our proposed simulation approach. Then, as illustrated in Fig. S3 and S4 (Supplementary Information), the points with the minimum and maximum PCE among the five points are located to be X_{min} and X_{max}. With X_{min} and X_{max}, the average point of the four points of the best side X_M is computed from $X_M = (sum(X_i)-X_{min})/4$. Then, the first possible better point is located by the reflection operation. The reflection point is obtained as $X_r = 2X_M-X_{min}$. If the PCE value (f(Xr)) for X_r is larger than that ($f(X_{max})$) of point X_{max}, the next possible better point is located through the expansion operation: the expansion point $X_e = 2X_r-X_M$. Then, if $f(X_e)>f(X_r)$, X_{min} is replaced by $X_{new} = X_e$; elese,

X_{new} = X_r. And if $f(X_r)<f(X_{max})$, the next possible better point is obtained through contraction operation, rather than expansion operation. The two contraction points is calculated as $X_{c1} = (X_M+X_{min})/2$ and $X_{c2} = 2X_M-X_{c1}$. If the larger PCE value of $f(X_{c1})$ and $f(X_{c2})$ is smaller than $f(X_{min})$, the possible better points are obtained by shrink operation; and all the five points are replaced as Xnewi = $(X_{max}+X_i)/2$, i = 1~5. Else if PCE value of X_{c1})is larger than $f(X_{min})$, X_{min} is replaced by X_{c1}. Else if $f(X_{c2})$ is larger than $f(X_{min})$, X_{min} is replaced by X_{c2}. This is one optimization iteration. Subsequently, this process is repeated again and again until the points converge and do not change any more. And the maximum PCE among the final five points are assumed to be the optimal PCE value for the system with the fixed $\eta_{P3HT:PCBM}$. For each value of $\eta_{P3HT:PCBM}$, the simplex searching algorithm is conducted to get the optimal PCE value.

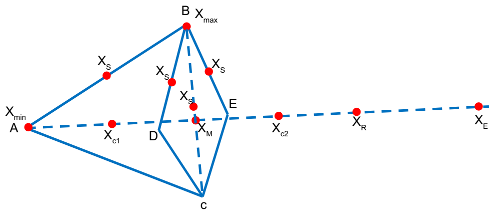

Figure S3: Schematic of the simplex searching algorithm. The detailed description of the simplex searching process is provided in the flow chart of this algorithm (Figure S2). A, B, C, D and E are the five starting points. X_{min} and X_{max} are the points with the minimum and maximum PCE, respectively. X_M is the average point of the four points of the best side. X_R is the point calculated in Reflection. X_E is the one acquired be Expansion. X_{c1} and X_{c2} are the two points obtained by Contraction operation. X_R are the points calculated according to the Shrink operation.

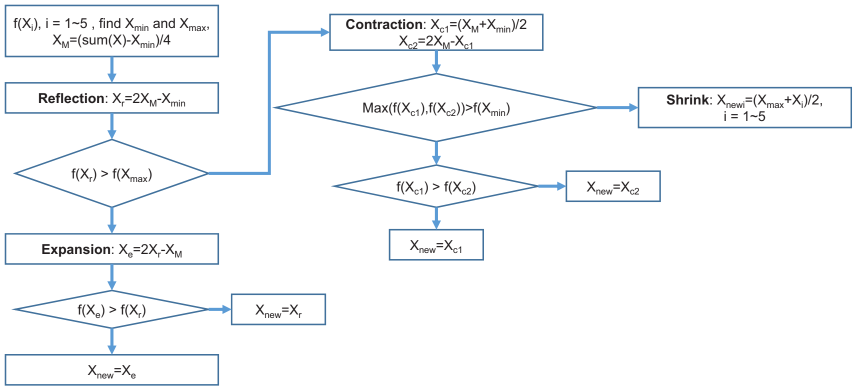

Figure S4: Flow Chart of the simplex searching algorithm applied in this paper.

4. Genetic algorithm for tandem polymer solar cells optimization

Similarly with simplex searching, for GA, six samples (points) are generated randomly within the investigated space. Each sample has four variables ($d_{P3HT:PCBM}$,

$d_{PCPDTBT:PCBM}$, $a_{P3HT:PCBM}$, $a_{PCPDTBT:PCBM}$, $\eta_{P3HT:PCBM}$). The PCE values for the six samples are computed through the proposed simulation method. And the two samples with the highest PCE values are chosen as parent samples to generate the child samples. The better one of the parent samples is reserved. By exchange traits (variables) of the two parent samples, the second child sample is generated. Next, each of the remaining four child samples are prepared by one mutation operation. In the mutation operation, one trait is changed to a random value. Then the new six samples are generated. This is one iteration of the optimization. This process is repeated again and again until the system achieves stable and the highest PCE value keeps constant. Then this PCE value is assumed to be the optimal PCE for the system. For each D/A weight ratio of P3HT:PCBM, we conduct the GA optimization and acquire the optimal PCE.

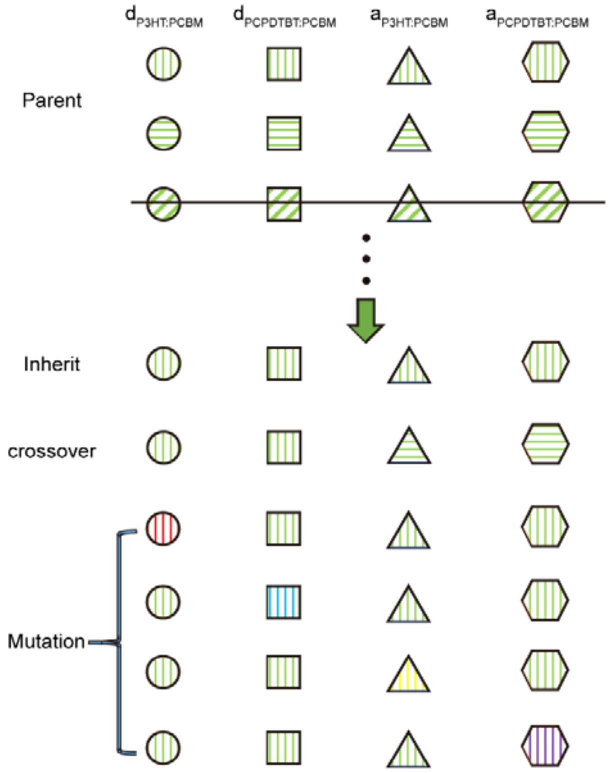

Figure S5: Schematic of GA algorithm used in this paper. Six samples are prepared. Among them, the two best ones are selected as parent samples. In the new samples, the best one in the former iteration is reserved. And then traits are exchanged between the best two ones to generate the second child sample. The next four child samples are prepared through mutation of each of the four traits.

5. Optimization testing results for the investigated tandem polymer solar cells

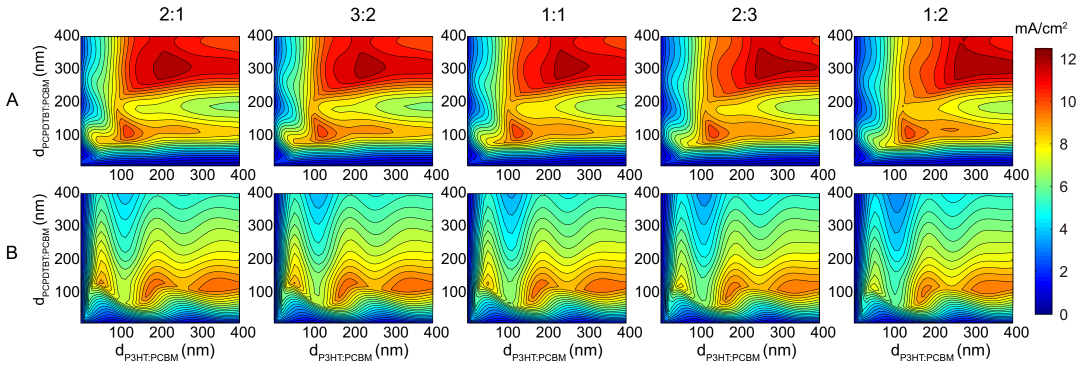

Figure S6: The possible maximum current density evaluated from optical calculation module for varied weight ratios and different configurations.

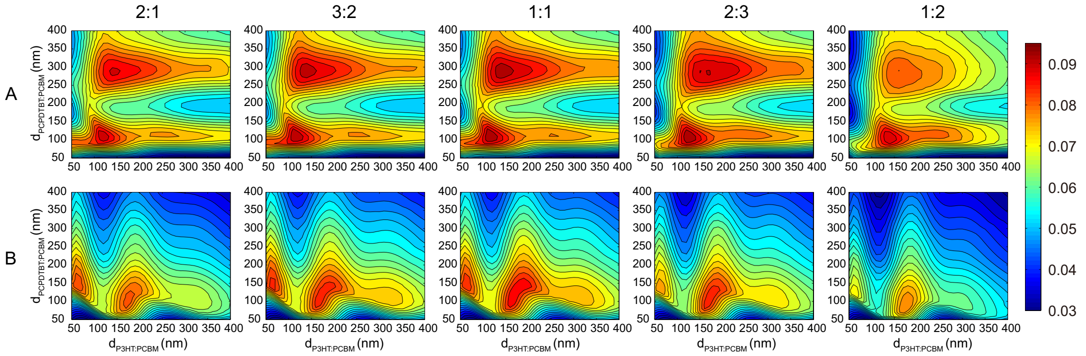

Figure S7: Maps of PCE with respect to thickness of the two active layers evaluated for the two configurations (A and B) and varied D/A weight ratios in P3HT:PCBM active layer. The average domain size is kept to be 10 nm for both the active layers.

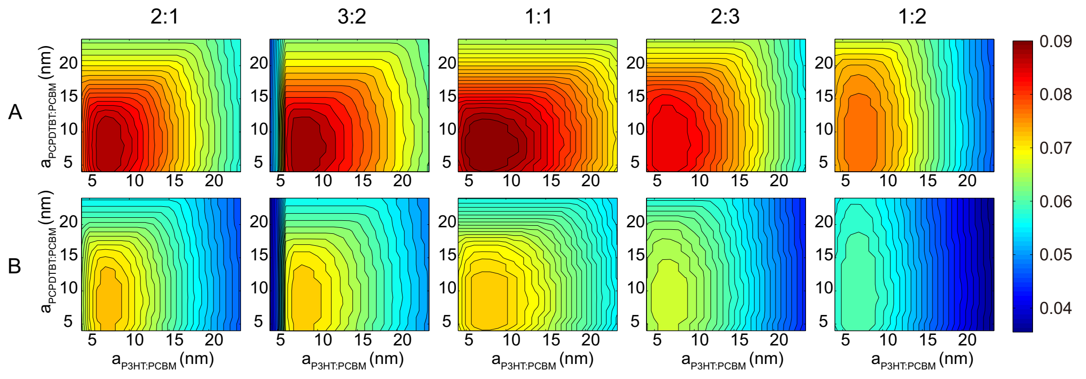

Figure S8: Maps of PCE with respect to average domain size of the two active layers evaluated for the two configurations (A and B) and varied D/A weight ratios in P3HT:PCBM active layer. The thickness is kept to be 100 nm for both the active layers.

6. References

1. Brush, S. G., *Reviews of modern physics* **1967,** *39* (4), 883.
2. Lei, B.; Yao, Y.; Kumar, A.; Yang, Y.; Ozolins, V., *J Appl Phys* **2008,** *104* (2), 024504.
3. Wei, F. N.; Liu, L. M.; Liu, L. Q.; Li, G. Y., *Ieee Journal of Photovoltaics* **2013,** *3* (1), 300-309.
